# Influence of *Codium tomentosum* Extract in the Properties of Alginate and Chitosan Edible Films

**DOI:** 10.3390/foods7040053

**Published:** 2018-04-01

**Authors:** Ana Augusto, Juliana R. Dias, Maria J. Campos, Nuno M. Alves, Rui Pedrosa, Susana F. J. Silva

**Affiliations:** 1 MARE—Marine and Environmental Sciences Centre, ESTM, Instituto Politécnico de Leiria, 2520-641 Peniche, Portugal; mcampos@ipleiria.pt (M.J.C.); nuno.alves@ipleiria.pt (N.M.A.); rpedrosa@ipleiria.pt (R.P.); susana.j.silva@ipleiria.pt (S.F.J.S.); 2 Centre for Rapid and Sustainable Product Development (CDRsp), Instituto Politécnico de Leiria, 2430-028 Marinha Grande, Portugal; juliana.dias@ipleiria.pt; 3 I3S-Instituto de Investigação e Inovação em Saúde, Universidade do Porto, 4200-135 Porto, Portugal; 4 INEB-Instituto de Engenharia Biomédica, Universidade do Porto, 4150-180 Porto, Portugal

**Keywords:** packaging materials, edible films, seaweeds, chitosan, alginate, natural, additive

## Abstract

The growing search for natural alternatives to synthetic food packaging materials and additives has increased, and seaweed extracts’ bioactivity has made them suitable candidates for incorporation in novel edible films. This study aims to investigate the effect of *Codium tomentosum* seaweed extract (SE) incorporation in alginate and chitosan edible films. Alginate- and chitosan-based films with and without the incorporation of 0.5% SE were characterized according to their physical, optical, mechanical, and thermal properties. Seaweed extract incorporation in chitosan films resulted in an increase of film solubility (50%), elasticity (18%), and decrease of puncture strength (27%) and energy at break (39%). In alginate films, the extract incorporation significantly decreased film solubility (6%), water vapour permeability (46%), and elasticity (24%), and had no effect on thermal properties. Depending on the type of application, the addition of SE in edible films can bring advantages for food conservation.

## 1. Introduction

In recent decades, the use of edible films as a bio-based food packaging has become increasingly relevant for researchers and the food industry. Edible films can act as a selective barrier to water, oxygen, and carbon dioxide transfer and solute movements, forming a thin layer of material between the food matrix and the environment. The incorporation of active substances within the film matrix allows additional product shelf-life extension [1,2], since film functionality can change with the nature of added components [3].

Chitin is the most abundant naturally occurring amino-polysaccharide, is a by-product from the crustacean industries, and its deacetylation produces chitosan. Chitosan is an edible and biodegradable polysaccharide with attractive characteristics—namely, high antimicrobial and antifungal activities [2,4,5]. Chitosan films are an environmentally friendly option for food packaging, and are known to have a high resistance to breakage and elasticity [6,7], two important characteristics for food packaging materials.

Alginate is a polysaccharide composed by glucuronic and mannuronic acid units extracted from brown seaweeds (class Phaeophyceae) and widely used in the food industry [3,8,9]. Alginate food applications are mainly based on the unique colloidal properties that allow its gel-forming ability through cation binding [9]. Despite the ability to form strong films, alginate films exhibit poor water resistance due to the material’s hydrophilic nature [10].

Edible seaweeds are a widespread and commercially valuable resource in food, fodder, and pharmaceutical industries, and as soil conditioner products [11]. They are also an important source of natural additives, such as antioxidants [12], antimicrobials, and polysaccharides [13]. Edible seaweeds are rich in bioactive metabolites that are not produced by terrestrial plants [14], and constitute a good source of natural additives for edible film incorporation. Despite the large number of studies in the literature regarding edible seaweed extracts [15], limited information exists concerning the extract of the edible seaweed *Codium tomentosum* (SE) [16,17,18]. A recent study showed that the immersion of minimally processed Fuji apple in a solution containing 0.5% *C. tomentosum* extract minimized apple browning in the refrigerated product for 20 days and inhibited peroxidase (POD) and polyphenoloxidase (PPO) activities [18].

The present work aims to assess how *C. tomentosum* extract incorporation in alginate and chitosan edible films influences the polymers’ barrier, optical, mechanical, and thermal properties. Alginate and chitosan films were selected, as these are widely used edible polymers in the food industry. The results of the present work are of relevant importance to determine the potential success of the incorporation of *C. tomentosum* seaweed extract in edible films, addressing public concerns to reduce the use of traditional food packaging, such as plastic films, and food waste, and longer food shelf-life. 

## 2. Materials and Methods

### 2.1. Chemicals

Alginic acid sodium salt, low viscosity was purchased from Alfa Aesar GmbH (Karlsrube, Germany) and used as received. Commercial chitosan from shrimp shells (≥75% deacetylation degree, Mw 50–190 kDa), 6-hydroxy-2,5,7,8-tetramethylchroman-2-carboxylic acid (Trolox), and 2,2′-azobis (2-methylpropionamidine) dihydrochloride (AAPH) were purchased from Sigma-Aldrich Co. (Steinheim, Germany).

### 2.2. Seaweed Collection and Seaweed Extract Preparation

Fresh *C. tomentosum* samples were collected in Peniche, Portugal in September 2012. Extractions were performed using water/ethanol as the extraction solvents [18]. The dried extract was stored at −80 °C (ultra-low freezer, Thermo Fisher Scientific, Waltham, MA, USA) until further analyses.

### 2.3. Extract Characterization

SE moisture content was determined with an automatic moisture analyser (model HB 43-S; Mettler Toledo, Giesen, Germany). Ash content was quantified following the procedures adopted by the Association of Analytical Communities (AOAC International) [19]. Nitrogen content was determined by the Kjeldahl method using a conversion factor of 6.25 (Kjeltech 2006, Foss Tecator, Hillerod, Denmark). Total lipid extraction method was achieved as previously described in the literature [20], following a dry matter basis. Carbohydrate content was estimated by difference of all other components. To estimate total phenolic content, the oxygen radical absorbance capacity assay (ORAC) of the seaweed extract was determined [21]. The ORAC value was calculated and expressed as micromoles of Trolox equivalents (TEs) per gram extract (µmol of TE g^−1^ extract) using the calibration curve of Trolox.

### 2.4. Film Preparation

Alginate film-forming solutions (FFSs) were prepared by suspending 1% (*w*/*v*) of alginate (A) in distilled water at 70 °C until complete dissolution. After cooling to 45 °C at room temperature, glycerol was added at 1% (*v*/*v*) of the total volume with gentle stirring for 15 min. The same procedure was used for the preparation of FFS with SE, but the addition of 0.5% (*w*/*v*) seaweed extract (AE) was made before alginate addition.

Chitosan (1% *w*/*v*) was dissolved into 1% (*w*/*v*) citric acid solution (C). The mixture was stirred continuously at room temperature for 8 hours to obtain a homogeneous solution. Tween 80 (0.1% *v*/*v*) was added as plasticizer after homogenization followed by filtration to the removal of undissolved material. FFS with SE incorporation was performed with the same procedure, but with the addition of 0.5% (*w*/*v*) seaweed extract (CE) before chitosan. C and CE were degassed prior to drying by keeping the solution in a vacuum oven for 3 h to remove the trapped air bubbles.

FFS were cast into dishes (Ø 120 mm) assuring a surface density of solids in the dry films of 70 g m^−2^ in all formulations and dehydrated in a tray dryer (Armfield Tray Drier Type Uop8-A, Ringwood, UK) until constant weight was reached (air flow rate of 0.70 m s^−1^, 23 °C, and relative humidity (RH) 55%). Dry films were preconditioned in desiccators (containing gel silica) at 20 ± 2 °C prior to testing.

### 2.5. Film Characterization

#### 2.5.1. FTIR-ATR

The Fourier transform infrared spectroscopy attenuated total reflection (FTIR-ATR) technique was used to evaluate the functional groups of the materials and to detect possible changes with the seaweed extract incorporation. The FTIR analysis was carried out using an Alpha-P FTIR-ATR spectrometer (Bruker Optik GmbH, Ettlingen, Germany) in a range of 4000–400 cm^−1^, at a 4 cm^−1^ resolution with 64 scans.

#### 2.5.2. Film Thickness and Light Absorption

All films were visually inspected for homogeneity. Films thickness was determined using a manual micrometer (Mettler Toledo Ltd., Leicester, UK) with an accuracy of 0.001 mm, and an average of 15 measurements taken at different locations in films was considered. 

Film light barrier properties were determined using transparency values (T) calculated using the film absorption, measured at 550 nm (A_550_) with a UV-160 UV-vis spectrophotometer (Thermo Electron Corporation, Waltham, MA, USA) and film thickness (mm) (x) [22]:

T = A_550_/x (1)

#### 2.5.3. Surface Color Measurement

Film colour was determined by a colorimeter Konica Minolta CR-400 (Minolta INC., Tokyo, Japan). The equipment was calibrated using a standard white reflective plate. CIELab scale was used with colour parameters expressed in terms of: L* (lightness), a* (red/green), and b* (yellow/blue). Nine measurements for each sample were taken, placing the film sample over the standard white plate (L* = 95.38; a* = −0.16; b* = 2.48). The Euclidean distance between two points was determined with the colour difference equation (ΔE), and whiteness index (WI) was calculated [23].

#### 2.5.4. Moisture Content and Film Solubility 

Film moisture content was determined with an automatic moisture analyser. Film solubility was determined by a gravimetric procedure [7,24]. The initial film dry weight (W_i_) and final dry weight (W_f_) were determined after a drying process at 105 °C for 24 h. Initial dried samples were immersed in 30 mL of distilled water and gently shaken for 24 h, followed by a drying process to determine W_f_. Film solubility (FS%) was calculated using the following equation:

FS % = (W_i_ − W_f_)/W_i_ × 100 (2)

#### 2.5.5. Water Vapour Permeability (WVP)

Film water vapour permeability was measured gravimetrically [7,25], with slight adaptations to hydrophilic edible films matrix. Films without defects were sealed to a cup mouth (cell) containing 100 mL of distilled water (100% RH, 2000 Pa vapour pressure at 23 °C) with an exposed film area of 6 cm^2^. Test cups with films were placed in contact with an atmosphere at 23 °C and 75.7% RH (2119.6 Pa vapour pressure); after 1 h for atmosphere adaptation, the cells were weighed (±0.0001 g) at intervals of 30 min during 4 h. The measured WVP (kg Pa^−1^ s^−1^ m^−1^) of the films was determined [7,23].

#### 2.5.6. Surface Film Wettability

The sessile drop method was used to estimate the surface hydrophobicity of the films. The water contact angle was determined to evaluate the film’s surface wettability, as well as the influence of SE extract on its properties. The static contact angle (θ) was measured with an optical tensiometer (Paralab Company, model Theta, Gondomar, Portugal) using water. 

Before measurements, samples were pre-conditioned at RH 0%. The tests were made at 23 °C within the first 10 s (12 Frames Per Second) after dropping the solvent (distilled water) onto film surfaces, to avoid variations due to solvent penetration onto the specimens. 

#### 2.5.7. Moisture Sorption Isotherms

Water vapour sorption isotherms were drawn based on the static method [24,26] by keeping film samples at 23 °C in desiccators with saturated salt solutions (MgCl_2_, KCl, Mg(NO_3_)_2_, NaCl, and KNO_3_) with water activity (*a*_w_) ranging from 0.330 to 0.930, until equilibrium was reached. Moisture content at equilibrium was determined by drying the film at 105 °C for 48 h.

#### 2.5.8. Mechanical Properties

Puncture tests were run as reported in literature [27] using a texture analyser Model TA.XT.plus (Stable Micro Systems, Surrey, UK) controlled by the Texture Exponent Software 32 (Stable Micro Systems, Surrey, UK). A cylindrical puncture probe (2 mm diameter; Stainless P/2) was used to determine the percentage of elongation at break. Nine replicates of each film were performed.

#### 2.5.9. Thermal Analysis

Thermal properties and stability were determined using the Simultaneous Thermal Analyser, STA 6000 system (PerkinElmer, Boston, MA, USA). For this, 3–4 mg samples were placed in ceramic pans and tested under dry nitrogen purge (flow rate of 20 mL min^−1^). Samples were submitted to temperature of 30 °C to 350 °C at a rate of 10 °C min^−1^. Melting endotherm peaks and peak areas were used to determine melting temperatures (Tm) and enthalpies of fusion (∆H_m_), respectively. Indium and silver samples were used as calibration standards.

### 2.6. Data Statistical Analysis

All measurements were performed in triplicate, except when stated otherwise. One-way analysis of variance (ANOVA), followed by Fishers Least Significant Difference (LSD) test for multiple comparisons of group means were applied to determine significant differences between films (A, AE, C, and CE). All data were checked for normality and homoscedasticity. This procedure was applied for all measurements under study. Where applicable, results are presented as mean ± standard deviation (SD). For all statistical tests, the significance level was set at *p* ≤ 0.05. All calculations were performed with IBM SPSS Statistics 21 (IBM, New York, NY, USA). 

## 3. Results and Discussion

### 3.1. C. tomentosum Extract Characterization

The proximal composition of *C. tomentosum* extract is shown in Table 1. The relatively higher ash proportion in seaweed extract (SE) (approx. 74%) can largely be accounted for by the lower moisture content, which can probably be attributed to the hygroscopic nature of the SE [28]. 

The peroxyl scavenging activity of *C. tomentosum* extract—as measured by the ORAC assay—was approximately 100 times lower than the values presented in other studies [16] when methanol and dichloromethane were used as extraction solvents. However, the use of extraction solvents for food applications has legal restrictions, and ethanol is one of the authorized solvents according to the European Directive 2009/32/EC [29].

### 3.2. Thickness, Surface Color, and Light Absorption 

The thickness of the formulated films ranged from 0.03 to 0.05 mm (Table 2). Alginate and chitosan films were transparent and homogeneous, and the incorporation of the SE resulted in heterogeneous regions in both films. Under high humidity conditions (e.g., laboratory facilities), alginate films absorbed moisture, becoming difficult to handle.

The optical properties of films are an important indicator of film suitability as an edible coating, as it interferes with the product appearance and may lead to consumer rejection [23,30]. The results of the film colour measurements are shown in Table 2. All films were transparent with a slight yellowish colour, in agreement with the measured b* value. The addition of SE resulted in increased b* values for both types of film (*p* < 0.05). Previous studies mentioned similar results, such as an increase in b* values in fish gelatin films with the incorporation of SE [31]. Red/green (a*), yellow/blue (b*) coordinates, and total colour differences significantly increased (*p* < 0.05) with the incorporation of SE in both alginate and chitosan-based films. The a* value has been used as a physical parameter to represent greenness in colour measurements [32]. Chlorophylls—the pigments responsible for the characteristic green colour of plants and which are present in *C. tomentosum*—are most likely responsible for the observed variation in a* value. Colour changes in the resulting film may be due to pigments remaining in the SE. As a consequence of L*, a*, and b* changes, the whiteness index values decreased with the addition of SE (*p* < 0.05).

The addition of SE to alginate films increased *t* values (*p* < 0.05), which indicates a lower degree of film transparency, corresponding to higher light absorption and consequently a higher film opacity [29,33]. On the contrary, the addition of SE to chitosan films decreased *t* values (*p* < 0.05), increasing film transparency appearance (Table 2).

### 3.3. FTIR-ATR Analysis

**Figure 1:**
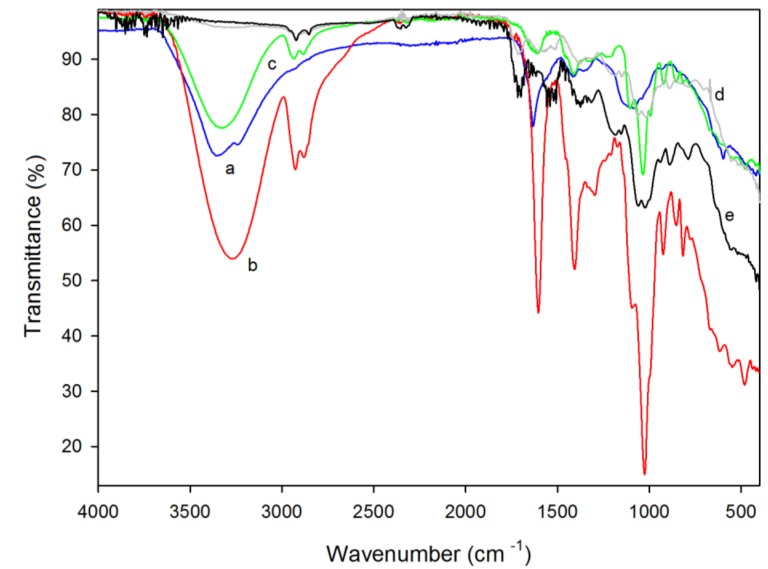
Fourier transform infrared spectroscopy attenuated total reflection (FTIR-ATR) spectrum of the (**a**) *C. tomentosum* seaweed extract (blue line) and the different tested films: (**b**) 1% alginate (red line), (**c**) 1% alginate with 0.5% of *C. tomentosum* seaweed extract (green line), (**d**) 1% chitosan (grey line), and (**e**) 1% chitosan with 0.5% of *C. tomentosum* seaweed extract (black line).

Through the interpretation of films’ FTIR spectra, it is possible to identify specific functional groups and therefore investigate possible interactions between the polysaccharides (alginate or chitosan) and the incorporated SE. FTIR spectra of the different formulated films and the seaweed extract (SE) are presented in Figure 1.

The FTIR spectra of *C. tomentosum* extract (Figure 1a) showed absorption bands at 1100–930 cm^−1^, which are normally present in seaweed polysaccharide standards and can represent both C-C and C-O pyranoid ring stretching and C-O-C glycosidic bond stretching [28]. The presence of proteins in the SE could be explained by the above-mentioned bonds, typically present in protein spectra [34]. As seen in Table 1, SE had 2% protein. SE showed a strong transmission band at 1630 cm^−1^, related to the stretching vibration of the (NH) C=O group—a group also observed in extracts of *Codium capitatum*, a seaweed from the same genus of *C. tomentosum* [34]. In these spectra, it is also possible to observe a broad band at 3357 cm^−1^ that could be related to the presence of polysaccharides in SE. The possible presence of the sulphated polysaccharide fucoidan in the SE could be associated to the sulphate (SO_4_) and methyl (CH_3_) group bands signals observed at 1414 cm^−1^ and 1360 cm^−1^, respectively [35,36]. The use of fucoidan as a nutraceutical and food supplement is under study, and therefore the presence of this polysaccharide in *C. tomentosum* extract can be an advantage—mostly due to its antioxidant and antibacterial properties [35].

The alginate film exhibited four outstanding bands corresponding to a COO- (asymmetric) stretching at 1603 cm^−1^, a COO- (symmetric) stretching band at 1407 cm^−1^, and a C-O-C stretching band at 1025 cm^−1^ (Figure 1b) [8,37] and the presence of bands at 817 cm^−1^ on the alginate films spectra, which indicates the presence of mannuronic acid [37].

A broad band at 3273 cm^−1^ representing hydroxyl groups (HO-) was also observed. The -CH vibration band occurs at 2928 cm^−1^, which can be overlapped with the COO- vibration bands [8,38]. No significant differences were observed between the FTIR spectra of alginate film with and without seaweed extract in terms of wavenumber absorbance (Figure 1c). A slight reduction of the bands size in the former bands was observed with SE incorporation. In both spectra, it was possible to identify the vibration bands from COO, CH, C-O, OH, and C-O-C groups.

The FTIR spectrum of the chitosan film (Figure 1d) showed a C-H stretching between 2922 cm^−1^ and 2920 cm^−1^ and bands at 1186 cm^−1^ and 1017 cm^−1^, indicating the presence of a free amino group at the C_2_ position of glucosamine (a major group present in chitosan). The presence of the first C-H stretching was also observed in chitosan films with seaweed extract (Figure 1e) at the same range of wavenumber; the second range of bands were also present, but with a minor intensity. The absence of a broad band around 1610 cm^−1^ (representing acetylated amino groups) in both C and CE spectra is associated with a high degree of deacetylation, which agrees with sample specifications (≥75% deacetylation degree) [39]. A broad band at 1712 cm^−1^ might be related to carbonyl vibration of the carboxylic acid [39]. 

SE interactions with alginate and chitosan matrices were mainly reflected in the bands’ areas, which represent the extent of interaction between them. In all cases, the addition of SE led to a variation in area, reflecting different intensities of the chemical bonds established in these materials. These differences may influence the following described film properties. 

### 3.4. Moisture Content and Film Solubility 

Film hydrophobicity is related to the amount of water present in films; the more hydrophilic the film, the higher the moisture content [23]. Table 2 shows the moisture content of the studied edible films. Alginate-based films showed the highest values of moisture (24–28%), with chitosan-based films presenting significantly lower moisture contents (9–10%) (environment RH of 75%).

Film solubility determines the biodegradability of films when used as food packaging, as well as their functionality as a water barrier [5]. Higher water solubility indicates lower water resistance. A and AE films (alginate-based films) showed the highest values of water solubility (86–90%) (Table 2), indicating the high hydrophilic character of alginate films in the presence of water (also reported in literature [23]), and lower film integrity in high-humidity environments [10]. The addition of SE to alginate films resulted in a decrease in water solubility (6%) (*p* < 0.05) (Table 2), perhaps due to the presence of hydrogen bond and hydrophobic interaction of protein in the film matrix [31]. Studies also showed that the extract of the seaweed *Turbinaria ornata* decreased the solubility of fish gelatin-based films [31], presenting lower values of solubility when compared to alginate-based films in the present study. As shown in Table 2, chitosan film had low water solubility, which was expected given that chitosan is mainly soluble in organic solutions and in acids such as hydrochloric, phosphoric, and nitric acid at pH bellow 6.5 [40]. The incorporation of SE into chitosan films resulted in a significant increase of water solubility (*p* < 0.05); researchers also reported an improvement of the water solubility of chitosan films with the incorporation of tea extracts [41]. In this study, the interaction between chitosan and SE could induce a decrease in the cross-linking degree of intermolecular chains in the matrix, thus resulting in the observed higher solubility. 

### 3.5. Water Vapor Permeability (WVP)

Films’ WVP at a relative humidity gradient of 100:75 are shown in Table 2. Measured values ranged from 0.514–1.22 × 10^−16^ kg Pa^−1^ s^−1^ m^−1^. The WVP values of the analysed alginate films were lower than the values presented in other studies [3,42]. These differences might be caused by different film preparation techniques (concentration and drying technique) and WVP measuring conditions (different RH gradient). The addition of SE caused a 45% (*p* < 0.05) reduction in the WVP of alginate films, possibly as a result of changes in the degree of crosslinkage that led to a reduction in the polymeric chain mobility. This effect was also reported in alginate films with higher concentrations of calcium [42]. Similar behaviour of gelatin films with *T. ornata* extract inclusion was observed in previous works [31], possibly due to the presence of phenolic compound in the SE that might enhance the crosslinkage of gelatin. SE incorporation seems to interfere in the hydrophilic portion of films, and consequently in the hydrophilic/hydrophobic ratio, affecting water vapour transfer that generally occurs in the hydrophilic zone of the films structure [43]. The results also showed that the incorporation of SE did not significantly influence the WVP of chitosan films (*p* > 0.05). Furthermore, WVP values were in the range of WVP obtained for similar films (WVP rate of 1.53 g h^−1^ mm^−1^ m^−1^ kPa^−1^), reflecting the low water barrier characteristics of chitosan films [44]. The obtained values for WVP were far from the ones presented by petroleum-based polymers commonly used in food packaging, which have a WVP rate of 9.14 × 10^−13^ g m^−1^ s^−1^ Pa^−1^ [44], indicating that the studied films still need further improvements if they are to be used as an alternative to these materials.

### 3.6. Surface Film Wettability

Water contact angle is an indicator of film surface hydrophilicity; the lower the contact angle, the greater the material surface hydrophilicity [45]. The contact angle between water droplets and the surfaces of alginate and chitosan films were analysed (Table 2). Chitosan films (C) showed a higher contact angle (θ = 78°) compared with the remaining films. Influences of SE incorporation on contact angle values was verified, with a significant decrease (*p* < 0.05) of values in both types of films. The use of a polar solvent (water, relative polarity of 1) and a moderately polar solvent (ethanol, relative polarity of 0.654) [46] in the extraction can lead to the extraction of polar compounds. The SE incorporation in alginate and chitosan increased the hydrophilicity and consequently the film wettability, justifying the contact angle of 8° in AE films and 21° in CE films. Even though SE incorporation led to films with high hydrophilicity and concomitant increase in WVP, the application of this type of solution in food matrixes can be efficient, as high values of film wettability are related to a high surface coating capacity, enabling easier application on the food surface [45].

The contact angle results were in good agreement with the obtained moisture values (Table 2); higher moisture content led to lower contact angles, indicating a greater ability to absorb water and thus explaining the high hydrophilicity.

### 3.7. Moisture Sorption Isotherms

**Figure 2:**
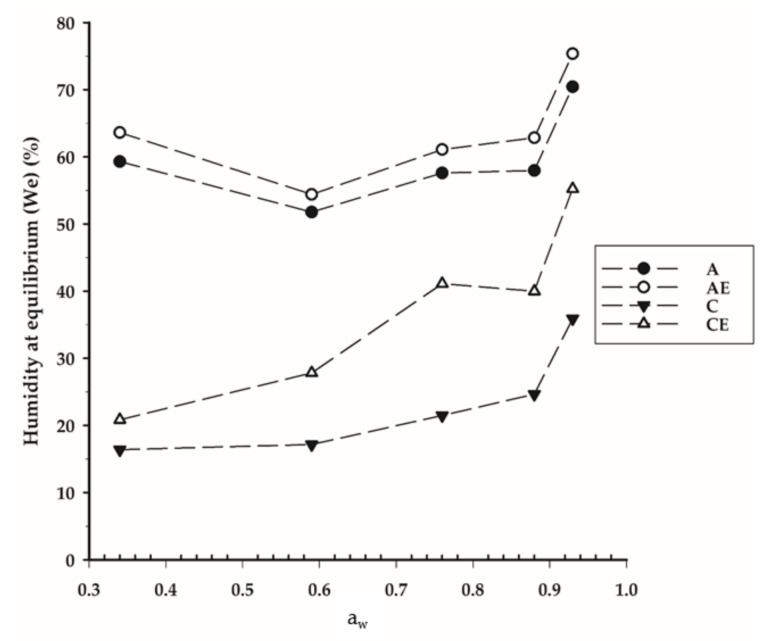
Moisture sorption isotherms of alginate without (A) and with seaweed extract (AE), chitosan without (C) and with extract (CE) films at 21 ± 2 °C. Data shown are the means (*n* = 3). *a*_w_: water activity.

Sorption isotherms of the films are presented in Figure 2 (0.34 to 0.94 *a*_w_ ranges). The initial moisture content of each film was different (Table 2), which means that the driving force of the sorption process was different, resulting in distinct curves. Higher values of moisture at equilibrium in alginate-based films were expected, since alginate is a hydrophilic polymer prone to absorb water vapour present in the atmosphere. High relative humidity values can have a negative effect on the application of alginate based films in food packaging systems, as it can lead to film solubilisation, causing failures in its structure and compromising its barrier properties [3]. Chitosan-based films (C and CE) presented lower moisture values at equilibrium since chitosan had a low water solubility, preventing water retention in the film and resulting in lower moisture values.

The incorporation of SE had no effect on the sorption isotherm of alginate-based film. On the contrary, in chitosan films, SE incorporation resulted in higher equilibrium moisture for all *a*_w_ tested in chitosan based films. This observation agrees with the results regarding the effect of SE in chitosan films, where extract incorporation significantly increased the film solubility and decreased contact angle (Table 2). 

### 3.8. Mechanical Properties

The mechanical properties of the studied films are presented in Table 3. Chitosan films with SE showed the highest elongation at break when compared to other film formulations, indicating higher film extensibility and mechanical strength. Extensibility and mechanical strength are two important material characteristics, related to the capacity of films to tolerate external stress and maintain integrity and barrier properties when applied as food packaging [7]. The addition of SE to chitosan films increased the elongation at break (*p* < 0.05), decreasing the puncture strength (*p* < 0.05) and energy at break (*p* < 0.05). These results were in contrast with those presented in other studies, where the elongation at break significantly decreased with the addition of antimicrobial compounds [41] and tea extracts into chitosan films [47]. These changes in mechanical properties could indicate that film structure softens, forming a more flexible film and consequently higher elongation values when subjected to tension and mechanical stress. SE could act as a plasticizer in film formulation, increasing the molecular mobility of the polymers [48] and resulting in the increase of elongation at break and the decrease of puncture strength. Mechanical properties of CE films were in agreement with the results of moisture and film solubility (Table 2). The incorporation of SE in alginate films led to a 23% decrease of elongation at break values (*p* < 0.05), increasing puncture strength and energy at break values. In this case, the incorporation of SE had the opposite effect of when applied on chitosan films, resulting in a film of higher brittleness. An equilibrium between the degree of polymer crosslinking and seaweed extract addition is required for better film workability characteristics, which changes film properties such as the solubility in water, affecting film brittleness (observed in Table 2) [48].

### 3.9. Thermal Properties

The thermal properties of the tested alginate- and chitosan-based films and SE are summarized in Table 3. At the temperature ranges of 27–100 °C for films and 75–115 °C for SE, it was possible to verify the first endothermic peak that could be associated with water loss [49,50]. The peaks in the range of 155–230 °C, 152–200 °C, and 131–153 °C were attributed to melting transition in alginate and chitosan based films [8,50,51] and SE, respectively. The melting temperature (Tm) of alginate and chitosan films was not affected by the addition of SE (*p* > 0.05).

SE incorporation resulted in a significant increase in degradation temperature (Td; *p* > 0.05) for both polymers (21 °C in alginate, 23 °C in chitosan). The same trend was observed in the crosslinking process of alginate films with *Aloe vera* [8]. Increasing degradation temperature is related to a higher thermal stability of films. SE incorporation in films resulted in an increase of melting points and temperature of degradation, indicating that these films may present more regular structures and higher packing capability or stronger inter-chain attraction [52]. This could be also correlated with the decrease of elongation at break (%) of AE films (Table 3), indicating the presence of a rigid structure.

Despite increased thermal stability due to the incorporation of SE, in chitosan films the melting enthalpy (ΔH_m_) values of the samples were lower (*p* < 0.05) when compared with the original films (Table 3). The decrease of ΔH_m_ values could reflect requirements of low energy to break the bonds (corroborated by the measured puncture strength and energy at break for different films shown in Table 3) when the sample was subjected to programmed heating, indicating a weak interaction between chitosan and SE [39]. 

The results of the thermal analysis of films are shown in Table 3. It was possible to observe two different steps: the first one at 86 °C could be associated with the devolatilisation of the sample, and the second one could be associated with the melting temperature [53]. This pattern can be related with crystalline solids that exhibit a pseudopolymorphic behavior—a term that refers to crystalline forms with solvent molecules as an integral part of the structure [54]. To verify the presence of solvent molecules in the extract structure, it would be necessary to carry out more studies, such as differential scanning calorimetry (DSC) associated with gas chromatography or mass spectrometry. The volatilization of the sample could be also observed in CE films (Td = 85 °C), revealing a possible crosslinking process between chitosan and SE.

## 4. Conclusions

The addition of *C. tomemtosum* extract to alginate and chitosan films significantly affected their mechanical integrity and barrier properties. The incorporation of *C. tomentosum* SE in alginate films showed the potential to decrease their solubility and WVP, which would render a better scope of applications in the food industry, allowing the possibility of its application in products with higher moisture content (e.g., minimally-processed fruits). On the other hand, chitosan films with *C. tomentosum* SE were easier to prepare due to solubility increase and showed greater flexibility and resistance to mechanical forces, which may indicate the possibility of its use as a film in products with more flexible properties.

## 5. Patents

Portuguese National Patent No. 107369. “Marine coating for application in minimally processed products or IV range products”. Augusto, A., Simões, T., Rodrigues, M.J., Campos, M.J., Leandro, S., Pedrosa, R., Silva, S.

## Figures and Tables

**Table 1:** Moisture, protein, ash, fat, and carbohydrate contents (% dry weight), and peroxyl radical scavenging activity (ORAC) of seaweed extract.

Test	Seaweed Extract
Moisture (%)	6.4 ± 1.2
Protein (%)	2.07 ± 0.06
Ash (%)	73.97 ± 0.33
Fat (%)	1.17 ± 0.06
Carbohydrate (%) ^a^	16.39
ORAC (Trolox equivalent, µmol g^−1^ extract)	5.99 ± 0.07

Results are the mean ± standard deviation (SD) (*n* = 3). ^a^ Determined by difference of all other components.

**Table 2:** Colour parameters, colour difference (ΔE), whiteness index (WI), and transparency (T), thickness (mm), moisture (%), water solubility (%), water vapour permeability (WVP), and contact angle (°) of alginate and chitosan films with and without seaweed extract (SE).

Test	Alginate	Alginate + SE	Chitosan	Chitosan + SE
Thickness (mm)	0.05 ± 0.02	0.03 ± 0.01	0.05 ± 0.01	0.06 ± 0.01
L*	92.68 ± 0.68	92.87 ± 0.15	93.89 ± 0.32	93.65 ± 0.93
a*	−0.44 ^A^ ± 0.02	−0.53 ^B^ ± 0.03	−0.46 ^A,C^ ± 0.05	−0.66 ^D^ ± 0.11
b*	6.75 ^A^ ± 0.17	9.42 ^B^ ± 0.39	3.96 ^C^ ± 0.21	5.20 ^D^ ± 0.73
ΔE ^#^	5.09 ^A^ ± 0.42	7.40 ^B^ ± 0.40	2.13 ^C^ ± 0.33	3.36 ^D^ ± 0.85
WI	91.69 ^A^ ± 0.34	88.17 ^B^ ± 0.37	92.70 ^C^ ± 0.35	91.73 ^A,D^ ± 0.87
T	2.4 ^A^ ± 0.1	13.1 ^B^ ± 0.4	3.1 ^C^ ± 0.3	2.5 ^D^ ± 0.1
Moisture (%)	24.86 ± 0.01	28.29 ± 0.01	9.56 ± 0.01	10.06 ± 0.01
Film solubility (%)	90.68 ^A^ ± 1.79	86.13 ^B^ ± 1.06	23.83 ^C^ ± 2.96	45.84 ^D^ ± 2.72
WVP (×10^−16^ kg Pa^−1^ s^−1^ m^−1^)	0.939 ^A^ ± 0.317	0.514 ^B^ ± 0.26	1.18 ^A^ ± 0.0071	1.22 ^A^ ± 0.0085
Contact angle (°)	37.70 ^A^ ± 0.15	8.9 ^B^ ± 0.30	78.58 ^C^ ± 0.27	21.80 ^D^ ± 0.57

Data shown are the means (± SD) (*n* = 9 for colour parameters, *n* = 15 for thickness; *n* = 3 for the other tests). ^A,B,C,D^ show significant differences in each test (*p* < 0.05, one-way analysis of variance (ANOVA), least significant difference (LSD) test). ^#^ colour differences between the standard white plate and film samples (see Section 2.5.3).

**Table 3:** Mechanical properties of alginate and chitosan films with and without seaweed extract (SE). Differential scanning calorimetry (DSC) data of films and seaweed extract.

Test	Alginate	Alginate + SE	Chitosan	Chitosan + SE	Seaweed Extract
Elongation at break (%)	69.81 ^A^ ± 4.06	53.68 ^B^ ± 2.92	60.81 ^C^ ± 7.03	74.60 ^D^ ± 4.60	-
Puncture Strength (N mm^−2^)	0.023 ^A^ ± 0.007	0.029 ^A^ ± 0.006	0.127 ^B^± 0.048	0.093 ^C^ ± 0.027	-
Energy at break (N s mm^−3^)	0.69 ^A^ ± 0.43	1.39 ^A^ ± 0.94	4.51 ^B^± 2.24	2.76 ^C^ ± 1.09	-
Tm (°C) *	184.22 ^A^ ± 1.15	196.64 ^A,B^ ± 1.89	180.07 ^A,C^ ± 0.61	193.10 ^A^ ± 13.94	144.70 ± 0.38
ΔH_m_ (J g^−1^) *	33.28 ^A^ ± 4.31	2.45 ^B^ ± 0.10	6.91 ^B^± 1.98	6.20 ^B^ ± 3.13	26.96 ± 5.98
Td (°C) *	180.68 ^A^ ± 0.71	201.05 ^B^ ± 4.14	164.17 ^C^ ± 8.63	187.22 ^A,D^ ± 5.23	86.62 ± 0.64 135.49 ± 0.47

Data shown are the means (±SD) (*n* = 9 for mechanical properties, *n* = 3 for DSC data). ^A,B,C,D^ show significant differences in each test (*p* < 0.05, ANOVA, LSD test). * Tm: melting temperature; ΔHm: melting enthalpy; Td: degradation temperature.
